# Pharmacokinetics of Oral Cannabinoid Δ8-Tetrahydrocannabivarin and Its Main Metabolites in Healthy Participants

**DOI:** 10.3390/ph17121603

**Published:** 2024-11-27

**Authors:** Cristina Sempio, Jorge Campos-Palomino, Jelena Klawitter, Erica N. Peters, Laura MacNair, Mehdi Haghdoost, Marcel O. Bonn-Miller, Amy Harrison, Shanna Babalonis, Uwe Christians, Jost Klawitter

**Affiliations:** 1 Department of Anesthesiology, University of Colorado, Anschutz Medical Campus, Aurora, CO 80045, USA; jorge.campos-palomino@ucdenver.edu (J.C.-P.); jelena.klawitter@cuanschutz.edu (J.K.); uwe.christians@cuanschutz.edu (U.C.); jost.klawitter@cuanschutz.edu (J.K.); 2 Canopy Growth Corporation, One Hershey Drive, Smiths Falls, ON K7A 0A8, Canada; epetie@gmail.com (E.N.P.); marcel.bonn-miller@charlottesweb.com (M.O.B.-M.);; 3 Charlotte’s Web, 700 Tech Ct., Louisville, CO 80027, USA; 4 Department of Behavioral Science, University of Kentucky, Lexington, KY 40536, USA; babalonis@uky.edu

**Keywords:** cannabinoids, THCV, pharmacokinetic, metabolites

## Abstract

Background: Tetrahydrocannabivarin (THCV) is a phytocannabinoid commonly found in cannabis with potential pharmacological properties; however, its post-acute pharmacokinetics (PK) in humans have not been studied yet. THCV has two isomers, Δ9- and Δ8-THCV, which seem to have different pharmacological properties. We investigated the PK of the Δ8-THCV isomer after oral administration as part of a two-phase, dose-ranging, placebo-controlled trial in healthy participants. Methods: Participants (*n* = 21) were enrolled in six study sessions and randomly received the following doses of a medium-chain triglyceride (MCT) oil oral formulation of Δ8-THCV: placebo, 12.5 mg, 25 mg, 50 mg, 100 mg, and 200 mg. Plasma samples from 15 participants were collected up to 8 h after administration and were analyzed by a validated two-dimensional high-performance liquid chromatography–tandem mass spectrometry assay. The trial was registered on clinicaltrials.gov (NCT05210634). Results: After oral administration, 11-nor-9-carboxy-Δ8-THCV (Δ8-THCV-COOH) was the main metabolite detected. The median time-to-maximum concentration (t_max_) ranged 3.8–5.0 h across doses for Δ8-THCV and 4.6–5.3 h for Δ8-THCV-COOH. The maximum concentration (C_max_) and area under the concentration–time curve over the observation period (AUC_last_) appeared to be dose-linear. Median AUC_last_ increased 2.3- to 4.8-fold and 1.7- to 2.9-fold for Δ8-THCV and Δ8-THCV-COOH, respectively, every two-fold increase in the dose. The isomers Δ9-THCV and Δ9-THCV-COOH were detected in plasma, despite being undetected in the formulated drug product analyzed by a third-party laboratory. Conclusions: For the first time, we report the pharmacokinetics of Δ8-THCV and its major metabolites after oral administration in humans. Δ8-THCV AUC_last_ showed dose linearity but the observed possible conversion to the Δ9-THCV isomer should be further studied.

## 1. Introduction

*Cannabis sativa* L. contains over 120 different cannabinoids in various concentrations depending on strain, genetic make-up and growth factors [1,2]. The legal status of cannabis varies across the countries and in the United States among states, from illegal to completely legal. The cannabis plant most likely originated form the Afghanistan region, but it is now grown worldwide especially in indoor facilities to increase reproducibility of the extracted products [1,2]. Recent studies focused on the therapeutic applications of the main constituents of cannabis, Δ9-tetrahydrocannabinol (THC) and cannabidiol (CBD) [3]. However, several so called “minor” cannabinoids have shown unique pharmacological properties that could lead to new therapeutic options for a variety of indications [4,5]. To date, minor cannabinoids are understudied in the scientific literature, despite being legally available in the United States since the 2018 Farm Bill was signed [6]. The minor cannabinoid Δ9-tetrahydrocannabivarin (Δ9-THCV) is found in low quantities in most cannabis varieties and is the propyl side-chain homolog of Δ9-THC [7]. Despite structural similarities, Δ9-THC and Δ9-THCV have significantly different pharmacological and physiological properties [8]. Δ9-THC is recognized as a partial agonist of the CB1 receptor, while in vitro studies suggest that Δ9-THCV at low doses is an antagonist of the same receptor and at higher doses a weak agonist [6,9]. Although most published studies focused on Δ9-THCV, THCV has a positional isomer, Δ8-THCV, which differs by location of the double bond in the alicyclic ring. Δ8-THCV is commonly synthesized by chemical isomerization of hemp-derived CBDV [9]. Δ9-THCV binds to a variety of receptors and cation channels, including both cannabinoid receptors (CB1 and CB2), serotonin 1A receptor (5-HT1A), transient receptor potential ankyrin 1 (TRPA1), and a variety of transient receptor potential vanilloid members (TRPV1–4) [4,5,8]. Less is known about Δ8-THCV’s activity, but in vitro studies have shown that, at low doses, ∆9-THCV is an approximately two times more potent CB1 receptor antagonist than the ∆8 counterpart [8]. Bátkai et al. showed that Δ8-THCV and its metabolite 11-OH-Δ8-THCV activate CB2 receptors in vitro and Δ8-THCV may decrease oxidative stress and inflammation in preclinical studies via CB2 receptor activation [10]. Only limited data on the pharmacokinetics (PK)/pharmacodynamics (PD) of Δ9-THCV in humans are available in the literature [9,11,12] and even less, if any, on Δ8-THCV and its metabolites.

Peters et al. recently published a study investigating the safety and effects of purified Δ8-THCV in healthy participants [6]. During this two-phase, dose-ranging, placebo-controlled study, plasma samples were collected across study sessions and the aim of the present study was to investigate the PK of oral Δ8-THCV and its major metabolites 11-hydroxy-Δ8-THCV (11-OH-Δ8-THCV) and 11-nor-9-carboxy- Δ8-THCV (Δ8-THCV-COOH).

## 2. Results

Results on participants characteristics, safety and tolerability of the oral MCT oil formulation of Δ8-THCV are presented elsewhere [6]. The overall conclusion was that Δ8-THCV was safe and well tolerated.

**Figure 1:**
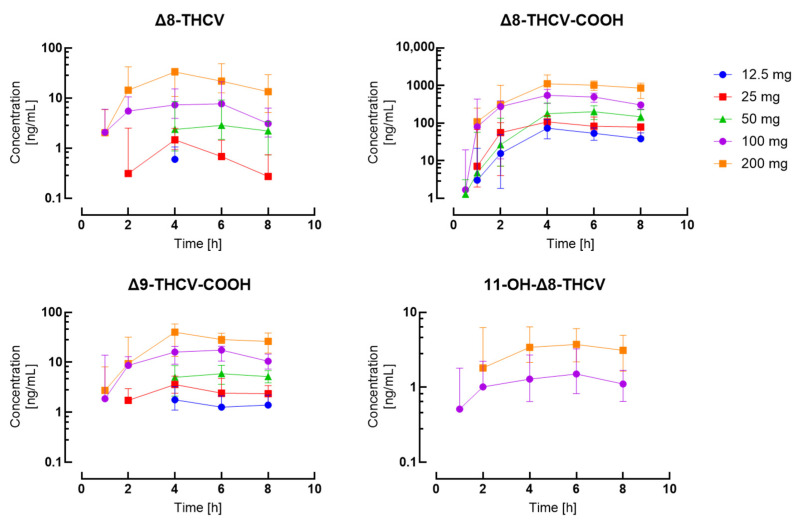
Comparison of pharmacokinetic profiles across doses for Δ8-THCV, Δ8-THCV-COOH, 11-OH-Δ8-THCV, and Δ9-THCV-COOH. Data are presented as median and interquartile ranges.

**Figure A1:**
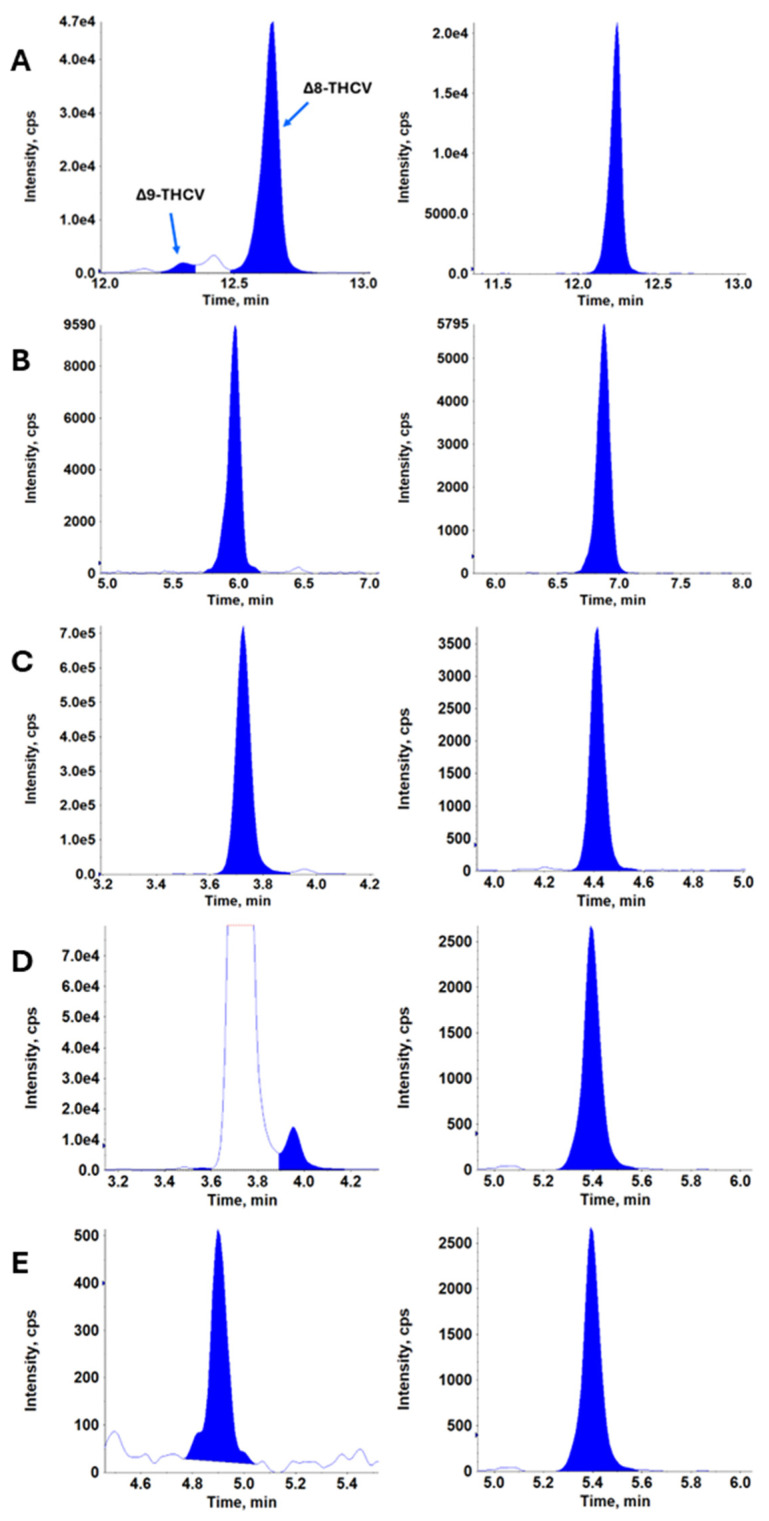
Representative extracted ion chromatograms of plasma samples from a subject after oral administration of highly purified Δ8-THCV in MCT oil. The left extracted ion chromatogram shows the analytes of interest, and the right extracted ion chromatogram shows the corresponding internal standards (IS). The concentrations for this sample were (**A**) Left Δ9-THCV 2.4 ng/mL, Right Δ8-THCV 69.8 ng/mL, IS Δ9-THCV-d5; (**B**) 11-hydroxy-Δ8-THCV 11.8 ng/mL, IS 7-hydroxy-CBD-d3; (**C**) Δ8-THCV-COOH 1026 ng/mL after 1:20 dilution, IS 7-CBD-COOH-d3; (**D**) Δ9-THCV-COOH 33.2 ng/mL, IS Δ9-THC-COOH-d3; and (**E**) Δ8-THC-COOH 1.4 ng/mL, IS Δ9-THC-COOH-d3.

A total of 504 plasma samples were collected from 15 participants after oral Δ8-THCV administration and 236 were positive for Δ8-THCV, 157 for 11-OH-Δ8-THCV, 381 for Δ8-THCV-COOH, 42 for Δ9-THCV, 260 for Δ9-THCV-COOH, and 49 for Δ8-THC-COOH. Δ9-THCV and Δ8-THC-COOH were present only after the 100 mg and 200 mg doses. No positive samples were detected for Δ9-THC, Δ8-THC, and their main metabolites, except for Δ8-THC-COOH. The results are summarized in Table A3, and Figure 1 depicts concentration–time curves for the different dose groups. Representative extracted ion chromatograms of plasma samples from a subject after oral administration of highly purified Δ8-THCV in MCT are presented in Figure A1.

**Figure 2:**
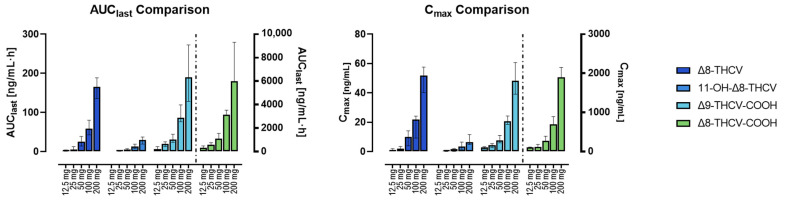
Comparison of C_max_ and AUC_last_ of Δ8-THCV, Δ8-THCV-COOH, 11-OH-Δ8-THCV, and Δ9-THCV-COOH across different oral doses of highly purified Δ8-THCV formulated in MCT oil. Data are presented as median and interquartile ranges. Δ8-THCV, 11-OH-Δ8-THCV, and Δ9-THCV-COOH refers to the left *Y*-axis; Δ8-THCV-COOH (separated by the dotted lines) to the right *Y*-axis.

Table 1 summarizes Δ8-THCV, 11-OH-Δ8-THCV, Δ8-THCV-COOH, Δ9-THCV, Δ9-THCV-COOH, and Δ8-THC-COOH pharmacokinetic results. Time to C_max_ (t_max_) for Δ8-THCV and 11-OH-Δ8-THCV occurred 1.3-fold later after doses greater than 50 mg compared to the 12.5 mg and 25 mg doses. The median t_max_ ranged 3.8–5.0 h across doses for Δ8-THCV and 4.6–5.3 h for Δ8-THCV-COOH. C_max_ and AUC_last_ showed dose-linearity for all compounds (Figure 2). Median AUC_last_ increased 2.3- to 4.8-fold and 1.7- to 2.9-fold for Δ8-THCV and Δ8-THCV-COOH, respectively, every two-fold increase in the dose. After the 200 mg dose, the median AUC_last_ ratio for Δ8-THCV-COOH/Δ8-THCV/11-OH-Δ8-THCV was 36:1:0.18. Due to the short plasma collection time (8 h), it was not possible to calculate the constant of elimination (k_el_), half-life (t_1/2_), volume of distribution (V_z_/F), and clearance (CL/F) for any of the compounds.

## 3. Discussion

The interest in therapeutic applications of the cannabis plant and in its major and minor cannabinoids, including Δ9-THC and Δ9-THCV isomers, has significantly increased over the past few years [10,11,13,14]. However, limited literature is available on the PK/PD of Δ9-THCV. Englund et al. administered oral Δ9-THCV to healthy participants for five consecutive days followed by an intravenous dose of THC; only 3 out of 37 plasma samples collected were positive for Δ9-THCV. Therefore, they were not able to report PK parameters [15]. Jadoon et al. administered oral doses of Δ9-THCV alone or in combination with CBD for 13 weeks to participants with type 2 diabetes, but no PK evaluation was performed [16]. Newmeyer et al. were the first to report PK parameters for Δ9-THCV and its major metabolite, Δ9-THCV-COOH, in occasional and frequent users after smoking, vaping, or oral consumption of standardized cannabis cigarettes/brownies that contained mainly THC [17]. Deiana et al. reported PK parameters in rats and mice after single oral or peritoneal administration of Δ9-THCV [18], while Moore et al. reported PK parameters in rats after oral gavage of six different doses of a mixture of 25.1% Δ8-THCV and 77.5% Δ9-THCV once daily for 14 days [19].

Elsohly et al. identified Δ9-THCV-COOH as the major metabolite of Δ9-THCV [20], and more recently, Rao et al. described several possible metabolites of Δ9-THCV including 11-OH-Δ9-THCV and Δ9-THCV-COOH [21]. To date, there are no studies assessing the metabolic pathway of Δ8-THCV and the human PK of the major Δ8-THCV metabolites, 11-OH- Δ8-THCV and Δ8-THCV-COOH have not been described. Our data confirmed that the Δ8-THCV metabolic pathway in humans corresponds to that of THC with the formation of the corresponding 11-hydroxy- and carboxy- metabolites. The same metabolic pathway could be suggested for Δ9-THCV with the formation of Δ9-THCV-COOH, but it could not be confirmed due to the lack of a certified reference standard for 11-OH-Δ9-THCV. Identification of the main metabolites of Δ8- and Δ9-THCV is pivotal to study their activity that is currently not well understood. Few studies analyzed the activity of Δ9-THCV [4,5,9,12] at different receptors but it is unclear if Δ8-THCV would have the same activity. Recent in vitro studies suggested that Δ9-THCV is a two-fold more potent antagonist at the CB1 receptor than Δ8-THCV [9] and that Δ8-THCV can activate CB2 receptors [10]. Furthermore, our data suggested that Δ8-THCV underwent a significant first-pass effect after oral administration. The plasma concentrations of Δ8-THCV-COOH were on average 10-fold higher at each timepoint than Δ8-THCV, while 11-OH-Δ8-THCV was consistently measured only after the two highest doses. Similarly, Newmeyer et al. did not detect Δ9-THCV in blood after oral administration, only Δ9-THCV-COOH [17]. Since the reference standard materials for Δ8-/Δ9- isomers of 11-OH-THCV recently became available, the present study is the first to describe the presence of 11-OH-Δ8-THCV in human plasma after oral administration.

Third-party analysis excluded the presence of Δ9-THCV in the Δ8-THCV MCT oil formulation used in the present study; however, Δ9-THCV and Δ9-THCV-COOH were detected in plasma. At present, the mechanism responsible for the observed conversion to Δ9-THCV is unclear and should be further investigated. PK parameters were calculated for these compounds. As aforementioned, Newmeyer et al. did not detect Δ9-THCV after oral administration, only Δ9-THCV-COOH [17]. Compared to our results, t_max_ for Δ9-THCV-COOH was shorter (3 h vs. 5 h) possibly due to the different oral formulation (brownie vs. MCT oil). The AUC calculated in occasional users for Δ9-THCV-COOH described by Newmeyer et al. [17] is similar to our Δ8-THCV-COOH AUC after the 12.5 mg dose in the present study.

In their safety and tolerability assessment of Δ8-THCV, Peters et al. observed increased ratings on Drug Effects Questionnaire (DEQ) items of “feel a drug effect” and “like the drug effect” and increases on the Addiction Research Center Inventory (ARCI) Marijuana scale at the two highest doses tested (100 mg and 200 mg) [6]. However, these THC-like effects did not correlate with impairment. Possible explanations for the increase in treatment-related effects could be the presence of increased concentrations of 11-OH-Δ8-THCV in plasma samples and the dose linearity observed. PK parameters for 11-OH-Δ8-THCV could be calculated only for the two highest doses but, to our knowledge, no studies have systematically assessed the activity of THCV metabolites in vivo. Only Bátkai et al. suggested that 11-OH-Δ8-THCV can activate CB2 receptors in vitro [10]. 11-OH-THC is known to be as active as its parent compounds THC [12,17]; therefore, considering the structural and metabolic similarities between THC and THCV, 11-OH-Δ8-THCV could potentially also be an active metabolite. Further investigations resulting in a better understanding of said metabolites’ contributions to Δ8-THCV’s activity are required.

## 4. Materials and Methods

The present clinical trial was conducted in accordance with consensus ethics principles, International Conference on Harmonization Good Clinical Practice guidelines, the Declaration of Helsinki, its amendments, and local laws and regulations. The protocol was approved by the Advarra Institutional Review Board (Pro00059879; approved 20 December 2021). The trial was registered on clinicaltrials.gov (NCT05210634). Written informed consent was obtained from each participant before any trial-related procedures were performed.

The study was a two-phase, dose-ranging, placebo-controlled trial in 21 healthy participants to assess the safety, tolerability, PK, and PD of an oral formulation of Δ8-THCV. A detailed description of the study was published elsewhere [6]. The study product was a medium-chain triglyceride (MCT) oil oral formulation of Δ8-THCV at a concentration of 50 mg/mL. Third-party analytical testing (ACS Laboratory, Sun City Center, FL, USA) had established Δ8-THCV concentration and purity (98.6%). Only cannabidiol (CBD, 0.5 mg/mL, 0.96%) and cannabigerolic acid (CBGA, 0.2 mg/mL, 0.44%) as other cannabinoids were present. Importantly, the absence of detectable Δ9-THCV and Δ8/Δ9-THC isomers was confirmed. MCT oil was used as placebo.

Twenty-one participants were enrolled in the clinical study and plasma samples from 15 participants were sent to our laboratory for pharmacokinetic evaluation. Of these, 3 participants were enrolled in Phase 1, an unblinded, placebo-controlled, single ascending dose design and 12 were enrolled in Phase 2, a double-blind, randomized, placebo-controlled, within-participant crossover design. At each study session, participants received one of the following oral doses: placebo, 12.5 mg, 25 mg, 50 mg, 100 mg, and 200 mg of the aforementioned Δ8-THCV formulation in MCT. Study visits were separated by a washout period of a minimum of one week and maximum of 28 days. Participants entered the research facility the night before the session and received the study product in the morning after a standardized high-fat, high-calorie breakfast. PK blood samples included in this analysis were collected prior to dosing and 0.5, 1, 2, 4, 6, and 8 h after dose. Immediately following collection into K_2_EDTA tubes, blood samples were placed on wet ice, centrifuged, and plasma was immediately frozen at −80 °C until shipment to the bioanalytical laboratory (iC42 Clinical Research and Development, University of Colorado, Aurora, CO, USA) on dry ice.

**Figure 3:**
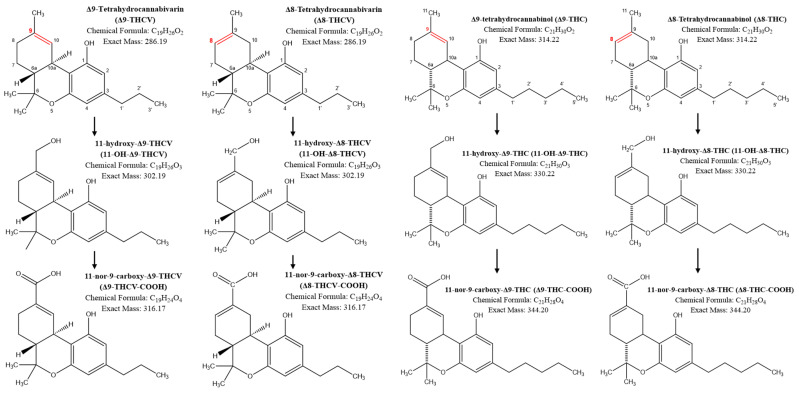
Major metabolic pathways of Δ9-tetrahydrocannabivarin (Δ9-THCV), Δ8-tetrahydrocannabivarin (Δ8-THCV), Δ9-tetrahydrocannabinol (Δ9-THC), and Δ8-tetrahydrocannabinol (Δ8-THC). The double bond is marked in red to highlight the difference between the Δ8- and Δ9-isomers.

Plasma samples were analyzed using a validated high-performance liquid chromatography–tandem mass spectrometry (LC-MS/MS) assay [12]. The assay included Δ8-THCV and its main metabolites 11-OH-Δ8-THCV and Δ8-THCV-COOH, Δ9-THCV and its main metabolite Δ9-THCV-COOH, and Δ9-THC and Δ8-THC as well as their main metabolites (Figure 3). The complete list of analyzed compounds and a summary of the key performance parameters of the assay are presented in Appendix A and Table A1 and Table A2. For the compounds of interest, the lower limits of quantification (LLOQ) were: 0.5 ng/mL for Δ8-THCV, 11-OH-Δ8-THCV, Δ9-THCV, Δ8-THC, and Δ9-THC and 1.0 ng/mL for Δ8-THCV-COOH, Δ9-THCV-COOH, and Δ8-THC-COOH. For the PK analysis, concentrations below the lower limit of quantification were set to 0 ng/mL. PK parameters were calculated using non-compartmental analysis (Phoenix WinNonlin version 8.4., Certara, Princeton, NJ, USA). Statistical analysis was carried out using GraphPad Prism (version 10, Boston, MA, USA).

## 5. Conclusions

In conclusion, this study is the first to characterize the pharmacokinetics of oral Δ8-THCV and its major metabolites, 11-hydroxy-Δ8-THCV and Δ8-THCV-COOH, in healthy participants. Our findings revealed that, in humans, Δ8-THCV undergoes a metabolic pathway similar to that of Δ9-THCV and THC, being primarily converted to 11-hydroxy and carboxy metabolites, with Δ8-THCV-COOH being the dominant metabolite in plasma. The results of the present study suggested substantial first-pass metabolism resulting in a 36-fold higher median systemic exposure (plasma AUC_last_) of Δ8-THCV-COOH than that of the parent compound. Δ8-THCV AUC_last_ after the different doses indicated dose linearity up to 200 mg. Additionally, the detection of Δ9-THCV and its metabolites, despite their absence in the study formulation, warrants further investigation into potential conversion mechanism. The present insights into the pharmacokinetics of Δ8-THCV provide a critical foundation for future research exploring its therapeutic potential and the activity of its metabolites in humans.

## Figures and Tables

**Table 1:** Summary of plasma pharmacokinetic parameters for the cannabinoids detected in human EDTA plasma after a single oral dose of 98.6% pure Δ8-THCV in MCT oil.

Timepoint	12.5 mg			25 mg			50 mg			100 mg			200 mg		
	*n*	Mean (±SD)	Median (Range)	*n*	Mean (±SD)	Median (Range)	*n*	Mean (±SD)	Median (Range)	*n*	Mean (±SD)	Median (Range)	*n*	Mean (±SD)	Median (Range)
**Δ8-THCV**															
t_max_	12	3.9 (±2)	4 (1–8)	14	3.8 (±1.8)	4 (1–6)	14	5 (±2)	6 (2–8)	14	4.2 (±2)	4 (1–8)	15	4.7 (±2.2)	4 (2–8)
C_max_	12	1.6 (±1.1)	1.1 (0.6–3.3)	14	2.6 (±1.7)	2.1 (0.9–5.7)	14	10 (±6.8)	10 (2.4–24.7)	14	21.5 (±13.3)	22 (5.7–50.4)	15	52.9 (±16.3)	51.7 (33.3–87.6)
t_last_	12	5.3 (±2.3)	5 (2–8)	14	6.3 (±1.9)	7 (4–8)	14	7.9 (±0.5)	8 (6–8)	14	8 (±0)	8 (8–8)	15	8 (±0)	8 (8–8)
C_last_	12	0.9 (±0.3)	0.7 (0.6–1.5)	14	1.1 (±0.4)	1 (0.5–1.8)	14	2.3 (±1.5)	2.2 (0.6–5.5)	14	4.1 (±3.1)	3.1 (0.8–9.9)	15	21.2 (±23.2)	13.4 (1.5–87.6)
AUC_last_	12	3.5 (±3.1)	2.1 (0.3–9.3)	14	7.4 (±5.5)	5.2 (1.4–16.5)	14	27.3 (±18.5)	24.9 (2.6–68.6)	14	65.5 (±34.3)	58.4 (15.7–137)	15	168 (±54.3)	165 (93.9–299)
MRT	12	4 (±1.9)	4 (1.4–8)	14	4.1 (±1.1)	4.2 (2.4–5.8)	14	5.2 (±1.4)	5.4 (2.5–8)	14	4.5 (±1.3)	4.1 (2.4–7.1)	15	4.7 (±1.3)	4.7 (2.7–6.5)
**11-OH-Δ8-THCV**															
t_max_	5	3.4 (±1.9)	4 (1–6)	7	3.7 (±1.8)	4 (2–6)	10	5 (±1.9)	6 (2–8)	14	4.2 (±2.4)	5 (1–8)	15	4.7 (±2)	4 (2–8)
C_max_	5	0.8 (±0.2)	0.8 (0.6–1.1)	7	0.9 (±0.2)	0.8 (0.6–1.3)	10	2 (±1)	1.9 (0.6–3.7)	14	4.3 (±3)	3.4 (1.3–10.6)	15	8.7 (±5.4)	6.4 (2.6–22.2)
t_last_	5	3.6 (±1.7)	4 (2–6)	7	4.3 (±1.8)	4 (2–6)	10	7.8 (±0.6)	8 (6–8)	14	7.6 (±1.2)	8 (4–8)	15	8 (±0)	8 (8–8)
C_last_	5	0.8 (±0.2)	0.8 (0.6–1.1)	7	0.8 (±0.1)	0.8 (0.6–1)	10	0.9 (±0.3)	0.8 (0.6–1.5)	14	1.4 (±0.9)	1.1 (0.5–3.8)	15	3.7 (±2.6)	3.1 (1.2–11.3)
AUC_last_	5	0.7 (±0.1)	0.8 (0.6–0.8)	7	1.5 (±1.9)	0.8 (0.4–5.8)	10	6.2 (±4)	5.3 (0.6–14.7)	14	13.9 (±9.9)	12.4 (1.5–40.3)	15	30.8 (±18.6)	29.5 (10.6–87.8)
MRT	5	1.5 (±1.7)	1.4 (0–4)	7	3.6 (±1.4)	3.6 (2–6)	10	5.7 (±1.5)	6.1 (3–8)	14	4.6 (±1.6)	4.3 (2.3–8)	15	4.8 (±1.2)	4.7 (3.2–6.6)
**Δ8-THCV-COOH**															
t_max_	15	4.9 (±1.7)	4 (2–8)	14	4.6 (±0.9)	4 (4–6)	14	5.3 (±1.3)	6 (4–8)	14	4.7 (±1.9)	4 (2–8)	15	5.3 (±1.8)	6 (2–8)
C_max_	15	99 (±37)	101 (39.2–178)	14	151 (±76.8)	116 (91.7–357)	14	324 (±165)	275 (114–765)	14	756 (±302)	699 (307–1392)	15	1738 (±579)	1899 (868–2594)
t_last_	15	8 (±0)	8 (8–8)	14	8 (±0)	8 (8–8)	14	8 (±0)	8 (8–8)	14	8 (±0)	8 (8–8)	15	8 (±0)	8 (8–8)
C_last_	15	46.8 (±23)	38.2 (20.1–102)	14	71.6 (±33.8)	78.3 (25.5–146)	14	166 (±60.3)	148 (78.9–264)	14	319 (±118)	304 (153–590)	15	917 (±582)	855 (364–2402)
AUC_last_	15	398 (±151)	331 (216–787)	14	642 (±298)	574 (264–1311)	14	1209 (±582)	1093 (364–2194)	14	3071 (±1101)	3132 (577–4951)	15	6955 (±2671)	5999 (3345–10759)
MRT	15	4.9 (±0.8)	4.9 (3.7–6.6)	14	4.8 (±0.6)	4.6 (3.8–5.9)	14	5.3 (±0.9)	5.3 (4–7.4)	14	4.8 (±1)	4.6 (3.6–6.7)	15	5.1 (±1)	5.1 (3.6–6.6)
**Δ9-THCV**															
t_max_	--	--	--	--	--	--	--	--	--	8	4.6 (±1.8)	5 (1–6)	13	4.9 (±2.3)	4 (2–8)
C_max_	--	--	--	--	--	--	--	--	--	8	1.1 (±0.5)	0.9 (0.5–1.9)	13	2 (±0.9)	1.7 (0.8–4.1)
t_last_	--	--	--	--	--	--	--	--	--	8	4.8 (±1.5)	5 (2–6)	13	6.2 (±2.1)	6 (2–8)
C_last_	--	--	--	--	--	--	--	--	--	8	1.1 (±0.5)	0.8 (0.5–1.9)	13	1.5 (±1)	1.4 (0.5–4.1)
AUC_last_	--	--	--	--	--	--	--	--	--	8	1.4 (±0.8)	1.2 (0.5–3.1)	13	4.8 (±2.9)	4.5 (1–10.2)
MRT	--	--	--	--	--	--	--	--	--	8	4.4 (±1.7)	4.4 (1.3–6)	13	4.5 (±1.6)	4.6 (2–7.2)
**Δ9-THCV-COOH**															
t_max_	15	5.1 (±2)	4 (2–8)	14	4.6 (±1.5)	4 (2–8)	14	5.1 (±1.9)	6 (2–8)	14	4.5 (±2)	4 (1–8)	15	5.3 (±2.1)	6 (2–8)
C_max_	15	2.7 (±1.2)	2.8 (1.1–5)	14	4.2 (±1.6)	4.4 (1.9–7.5)	14	8.8 (±3.7)	7.7 (3.7–16.1)	14	22.5 (±8.3)	20.6 (11.3–41.6)	15	50.3 (±13.8)	48.2 (31.4–71.1)
t_last_	15	6.8 (±1.7)	8 (4–8)	14	7.6 (±1.2)	8 (4–8)	14	8 (±0)	8 (8–8)	14	8 (±0)	8 (8–8)	15	8 (±0)	8 (8–8)
C_last_	15	1.9 (±0.8)	1.7 (1.1–3.3)	14	2.6 (±1)	2.4 (1.3–4.4)	14	5.4 (±1.9)	5.2 (2.4–8.5)	14	10.8 (±3.7)	10.5 (6.2–16.1)	15	29.2 (±16.1)	26.3 (10.9–63.8)
AUC_last_	15	7.9 (±5.7)	5.6 (1.1–20.1)	14	18.3 (±8.7)	19.6 (2.5–30.6)	14	33.4 (±15.8)	30.8 (8.7–62.7)	14	92.5 (±35.3)	86.5 (17.6–149.2)	15	201 (±77.9)	190 (89.7–336)
MRT	15	5.1 (±1.3)	5.3 (3.2–8)	14	4.8 (±0.7)	4.6 (3.9–6.1)	14	5.5 (±1)	5.5 (4–7.4)	14	4.9 (±1)	4.6 (3.7–7)	15	5.1 (±1)	5.2 (3.7–6.7)
**Δ8-THC-COOH**															
t_max_	--	--	--	--	--	--	--	--	--	8	5 (±1.1)	5 (4–6)	15	6.1 (±1.6)	6 (4–8)
C_max_	--	--	--	--	--	--	--	--	--	8	1.4 (±0.3)	1.4 (1–1.7)	15	2.4 (±0.7)	2.5 (1.4–3.8)
t_last_	--	--	--	--	--	--	--	--	--	8	5.8 (±1.3)	6 (4–8)	15	7.5 (±1.2)	8 (4–8)
C_last_	--	--	--	--	--	--	--	--	--	8	1.3 (±0.2)	1.3 (1–1.7)	15	1.9 (±0.6)	1.8 (1.1–2.8)
AUC_last_	--	--	--	--	--	--	--	--	--	8	2.4 (±1.5)	1.6 (1–4.7)	15	8.1 (±4.5)	9.6 (1.4–15)
MRT	--	--	--	--	--	--	--	--	--	8	5.2 (±1)	5.3 (4–6.5)	15	5.7 (±1.3)	5.8 (3.1–8)

No positive samples were detected for Δ9-THC and its main metabolites as well as for Δ8-THC and 11-OH-Δ8-THC. Abbreviations: Δ8-THCV: Δ8-tetrahydrocannabivarin, 11-OH-Δ8-THCV: (±)-11-hydroxy-Δ8-THCV, Δ8-THCV-COOH: 11-nor-9-carboxy-Δ8-THCV, Δ9-THCV: Δ9-tetrahydrocannabivarin, Δ9-THCV-COOH: 11-nor-9-carboxy-Δ9-THCV, Δ8-THC-COOH: (+)-11-nor- Δ8-THC-9-carboxylic acid, SD: standard deviation, C_last_: last quantifiable concentration, t_last_: time to last quantifiable concentration, MRT: mean residence time. The median time-to-maximum concentration (t_max_) ranged 3.8–5.0 h across doses for Δ8-THCV and 4.6–5.3 h for Δ8-THCV-COOH. The maximum concentration (C_max_) and area under the concentration–time curve over the observation period (AUC_last_) appeared to be dose-linear. Median AUC_last_ increased 2.3- to 4.8-fold and 1.7- to 2.9-fold for Δ8-THCV and Δ8-THCV-COOH, respectively, every two-fold increase in the dose.

## Data Availability

The datasets generated and analyzed during the current study are available from the corresponding author upon reasonable request.

## Appendix A

Summary of the key validation results of an LC-MS/MS assay to quantify Δ8-THCV, Δ9-THCV, Δ8-THC, Δ9-THC, and their respective metabolites in human plasma [12]. All corresponding isotope-labeled internal standards available at the time of method validation were included to improve assay performance. If a compound did not have a corresponding isotope-labeled internal standard, an internal standard with similar chemical structure and retention time was selected (Table A1).

**Table A1:** Lower limit of quantification (LLOQ) and analytical measuring range in human K_2_EDTA plasma.

Component	Internal Standard	LLOQ [ng/mL]	Analytical Measuring Range [ng/mL]
Δ8-THCV	Δ9-THCV-d_5_	0.5	0.5–400
Δ9-THCV	Δ9-THCV-d_5_	0.5	0.5–400
11-OH-Δ8-THCV	7-OH-CBD-d_3_	0.5	0.5–400
Δ8-THCV-COOH	7-CBD-COOH-d_3_	1.0	1.0–400
Δ9-THCV-COOH	Δ9-THC-COOH-d_3_	1.0	1.0–400
Δ8-THC	Δ8-THC-d_9_	0.5	0.5–400
Δ9-THC	Δ9-THC-d_9_	0.5	0.5–400
9S-Δ10-THC	Δ8-THC-d_9_	0.5	0.5–400
9R-Δ10-THC	Δ8-THC-d_9_	0.5	0.5–400
11-OH-Δ8-THC	11-OH-Δ9-THC-d_3_	1.0	1.0–400
11-OH-Δ9-THC	11-OH-Δ9-THC-d_3_	1.0	1.0–400
Δ8-THC-COOH	Δ9-THC-COOH-d_3_	1.0	1.0–400
Δ9-THC-COOH	Δ9-THC-COOH-d_3_	1.0	1.0–400

Abbreviations: Δ9-THCV: Δ9-tetrahydrocannabivarin, Δ9-THCV-COOH: 11-nor-9-carboxy-Δ9-THCV, Δ8-THCV: Δ8-tetrahydrocannabivarin, 11-OH-Δ8-THCV: (±)-11-hydroxy-Δ8-THCV, Δ8-THCV-COOH: 11-nor-9-carboxy-Δ8-THCV, Δ9-THC: Δ9-tetrahydrocannabiol, 11-OH-Δ9-THC: (±)-11-hydroxy-Δ9-THC, Δ9-THC-COOH: (+)-11-nor- Δ9-THC-9-carboxylic acid, Δ8-THC: Δ8-tetrahydrocannabiol, 11-OH-Δ8-THC: (±)-11-hydroxy-Δ8-THC, Δ8-THC-COOH: (+)-11-nor- Δ8-THC-9-carboxylic acid, 9S-Δ10-THC: (6αR,9S)-Δ10-Tetrahydrocannabinol, 9R-Δ10-THC: (6αR,9R)-Δ10-Tetrahydrocannabinol, 7-OH-CBD-d_3_: 7-hydroxy-cannabidiol-d3, 7-CBD-COOH-d_3_: 7-mor-7-carboxy cannabidiol-d_3_; LLOQ: lower limit of quantification.

**Table A2:** Intra- and inter-day imprecision and accuracy in K_2_EDTA human plasma. Intra-day *n* = 6/5 quality control (QC) levels; inter-day *n* = 18/5 QC levels.

Intra-Day Summary					Inter-Day Summary			
Component	Mean Accuracy	Accuracy Range	Mean Precision	Precision Range	Mean Accuracy	Accuracy Range	Mean Precision	Precision Range
Δ8-THCV	103.1	95.0–106.4	4.66	3.19–18.2	103.1	94.2–106.7	4.91	4.33–14.7
Δ9-THCV	97.1	80.3–105.2	9.96	1.68–6.78	95.8	82.9–100.7	7.64	4.55–12.3
11-OH-Δ8-THCV	100.8	96.4–104.1	2.90	3.91–9.69	99.0	96.3–102.7	2.58	5.09–12.4
Δ8-THCV-COOH	95.3	86.7–101.8	6.66	5.81–14.4	97.1	90.0–104.3	5.74	7.51–16.1
Δ9-THCV-COOH	97.1	93.2–100.0	3.22	5.90–18.3	96.0	91.6–99.7	4.16	7.80–17.5
Δ8-THC	105.1	102.7–109.9	2.66	4.09–18.5	105.6	101.6–108.3	2.34	5.65–15.5
Δ9-THC	101.6	98.2–106.6	3.05	4.38–14.5	99.5	97.9–101.6	1.35	5.78–11.3
Δ10-9S-THC	100.2	89.7–109.3	8.84	2.49–18.6	100.9	89.2–107.8	7.31	6.16–29.0
Δ10-9R-THC	102.5	88.9–110.2	8.81	3.76–43.0	103.5	91.6–108.8	7.08	4.78–27.1
11-OH-Δ8-THC	95.9	83.6–101.0	7.34	6.70–17.7	98.6	87.5–103.1	6.62	7.20–16.5
11-OH-Δ9-THC	106.7	100.5–119.2	6.88	4.32–23.3	101.8	95.7–105.2	3.82	4.71–25.3
Δ8-THC-COOH	104.5	99.8–114.1	5.42	3.58–15.6	100.4	96.9–103.5	2.66	6.24–15.7
Δ9-THC-COOH	99.8	96.0–102.0	2.47	3.37–15.5	96.1	88.8–99.4	4.72	5.97–12.4

Abbreviations: Δ9-THCV: Δ9-tetrahydrocannabivarin, Δ9-THCV-COOH: 11-nor-9-carboxy-Δ9-THCV, Δ8-THCV: Δ8-tetrahydrocannabivarin, 11-OH-Δ8-THCV: (±)-11-hydroxy-Δ8-THCV, Δ8-THCV-COOH: 11-nor-9-carboxy-Δ8-THCV, Δ9-THC: Δ9-tetrahydrocannabiol, 11-OH-Δ9-THC: (±)-11-hydroxy-Δ9-THC, Δ9-THC-COOH: (+)-11-nor- Δ9-THC-9-carboxylic acid, Δ8-THC: Δ8-tetrahydrocannabiol, 11-OH-Δ8-THC: (±)-11-hydroxy-Δ8-THC, Δ8-THC-COOH: (+)-11-nor-Δ8-THC-9-carboxylic acid.

Summary of the key performance parameters of the LC-MS/MS assay used to quantify the cannabinoids and their major metabolites in the present study (continued, please see reference [12] for more details).

*Specificity*: The response at the retention time of all compounds in the extracted ion chromatograms of blank matrix was less than 20% of the detector response of that of the lowest calibrator. Thus, the LC-MS/MS assay is considered specific in the tested matrix.

*Matrix effects*: No significant matrix effects (ion suppression/ion enhancement) were found in K_2_EDTA human plasma.

*Carry-over*: No relevant carryover was observed in plasma. Consistently, less than 0.25% carryover was found.

*Extraction recovery*: The mean recovery in K_2_EDTA human plasma was 83.9%.

*Stabilities*: All analytes were stable at the tested concentrations on the benchtop for up to 4 h. Extracted samples in the autosampler at +4 °C are stable for at least 72 h, and K_2_EDTA plasma samples containing all analytes could undergo at least three freeze–thaw cycles. All analytes were stable at the tested concentrations for up to 1 week at +4 °C, −20 °C, and −80 °C.

In general, the assay met acceptance criteria, and it was concluded that it was fit for analyzing study samples from pre-clinical studies in human plasma.

**Table A3:** Summary of plasma cannabinoids concentrations (ng/mL) by Δ8-THCV dose.

Timepoint	12.5 mg			25 mg			50 mg			100 mg			200 mg		
	BLOQ (*n* = 15)	Mean (SD, %CV)	Median (IQ Range)	BLOQ (*n* = 14)	Mean (SD, %CV)	Median (IQ Range)	BLOQ (*n* = 14)	Mean (SD, %CV)	Median (IQ Range)	BLOQ (*n* = 14)	Mean (SD, %CV)	Median (IQ Range)	BLOQ (*n* = 15)	Mean (SD, %CV)	Median (IQ Range)
**Δ8-THCV**															
Predose	15	0 (±0, --)	0 (0–0)	14	0 (±0, --)	0 (0–0)	14	0 (±0, --)	0 (0–0)	14	0 (±0, --)	0 (0–0)	15	0 (±0, --)	0 (0–0)
0.5 h	15	0 (±0, --)	0 (0–0)	14	0 (±0, --)	0 (0–0)	13	0.1 (±0.3, 374)	0 (0–0)	12	0.2 (±0.5, 254)	0 (0–0)	14	0.1 (±0.3, 387)	0 (0–0)
1 h	12	0.1 (±0.3, 212)	0 (0–0)	11	0.3 (±0.6, 238)	0 (0–0)	10	0.5 (±1, 201)	0 (0–0.5)	6	4.2 (±6.1, 145)	2.1 (0–4.9)	6	3.6 (±4.5, 124)	2 (0–5.2)
2 h	9	0.4 (±0.8, 184)	0 (0–0.7)	7	1.4 (±1.9, 140)	0.3 (0–2.1)	8	2.6 (±5.3, 204)	0 (0–1.4)	4	7.1 (±7.9, 113)	5.5 (0.2–8)	5	21.4 (±21.8, 102)	14.3 (0–36.6)
4 h	7	0.7 (±1.1, 145)	0.6 (0–0.9)	1	1.8 (±1.4, 78.9)	1.5 (1–2.3)	3	4.3 (±4.6, 109)	2.4 (1.3–6.8)	1	11.4 (±12.4, 109)	7.3 (4.6–11.6)	1	29.4 (±21.2, 72.2)	33.3 (12.6–38.6)
6 h	11	0.5 (±0.8, 178)	0 (0–0.7)	6	0.9 (±1.1, 120)	0.7 (0–1.4)	1	6.1 (±7.3, 120)	2.8 (1.7–7.3)	0	12.3 (±13.6, 110)	7.7 (2.8–18.5)	0	26.6 (±20.3, 76.2)	21.8 (12.9–40.9)
8 h	11	0.2 (±0.3, 172)	0 (0–0.3)	7	0.4 (±0.5, 117)	0.3 (0–0.7)	1	2.2 (±1.6, 71.4)	2.2 (0.8–3.2)	0	4.1 (±3.1, 74.8)	3.1 (1.8–5.9)	0	21.2 (±23.2, 109)	13.4 (5.3–28.3)
**11-OH-Δ8-THCV**															
Predose	15	0 (±0, --)	0 (0–0)	14	0 (±0, --)	0 (0–0)	14	0 (±0, --)	0 (0–0)	14	0 (±0, --)	0 (0–0)	15	0 (±0, --)	0 (0–0)
0.5 h	15	0 (±0, --)	0 (0–0)	14	0 (±0, --)	0 (0–0)	14	0 (±0, --)	0 (0–0)	14	0 (±0, --)	0 (0–0)	15	0 (±0, --)	0 (0–0)
1 h	14	0 (±0.1, 387)	0 (0–0)	13	0.1 (±0.2, 374)	0 (0–0)	12	0.1 (±0.4, 274)	0 (0–0)	7	1.4 (±2.2, 161)	0.5 (0–1.5)	8	1.2 (±1.5, 126)	0 (0–1.8)
2 h	13	0.1 (±0.3, 282)	0 (0–0)	10	0.3 (±0.4, 174)	0 (0–0.4)	12	0.4 (±1.1, 264)	0 (0–0)	4	1.8 (±2.3, 132)	1 (0.1–1.5)	5	4.5 (±5.7, 128)	1.8 (0–5.9)
4 h	13	0.1 (±0.3, 264)	0 (0–0)	11	0.2 (±0.4, 203)	0 (0–0)	9	0.6 (±0.9, 154)	0 (0–1.1)	3	2.1 (±2.8, 129)	1.3 (0.9–2.6)	1	5.2 (±5.4, 104)	3.4 (2.4–5.9)
6 h	14	0.1 (±0.2, 374)	0 (0–0)	11	0.2 (±0.3, 201)	0 (0–0)	5	1 (±1, 103)	0.9 (0–1.5)	2	2.4 (±2.3, 95.6)	1.5 (1–2.9)	0	4.6 (±2.9, 63.1)	3.7 (2.2–6)
8 h	15	0 (±0, --)	0 (0–0)	14	0 (±0, --)	0 (0–0)	5	0.6 (±0.5, 87.9)	0.6 (0–0.9)	2	1.3 (±1, 80.3)	1.1 (0.7–1.6)	0	3.7 (±2.6, 71.6)	3.1 (1.7–4.9)
**Δ8-THCV-COOH**															
Predose	13	0.2 (±0.4, 267)	0 (0–0)	13	0.3 (±1, 374)	0 (0–0)	10	0.8 (±1.6, 187)	0 (0–0.9)	10	0.5 (±0.8, 166)	0 (0–1)	14	0.1 (±0.3, 387)	0 (0–0)
0.5 h	12	0.4 (±0.9, 237)	0 (0–0)	9	2.7 (±5.2, 194)	0 (0–3.2)	6	4.7 (±10.4, 223)	1.3 (0–2.8)	5	13 (±22.1, 170)	1.7 (0–17.8)	10	5.6 (±16.2, 289)	0 (0–1.4)
1 h	5	15 (±22, 147)	3 (0–19.9)	2	28.5 (±35.7, 125)	7.1 (2.3–61.6)	4	27.3 (±42.3, 155)	4.8 (0.6–36.7)	2	202 (±229, 113)	79.5 (4–426)	3	158 (±231, 146)	108.5 (4.7–224)
2 h	2	30.1 (±34.2, 114)	15.5 (2.1–44.4)	0	54.6 (±48.9, 89.6)	55.6 (5.2–94.4)	1	84.1 (±118, 140)	26.5 (8.7–101)	0	306 (±371, 121)	275 (27.5–372)	1	653 (±732, 112)	318.8 (8.5–1009)
4 h	0	84.6 (±47.9, 56.7)	73.1 (47.9–116)	0	136 (±83.3, 61.1)	108 (94.1–143)	0	214 (±157, 73.2)	177 (93.5–307)	0	556 (±333, 59.9)	546.6 (439–755)	0	1302 (±838, 64.4)	1111 (530–1902)
6 h	1	62.2 (±39.8, 63.9)	53.3 (37.5–66.4)	0	96.3 (±50.9, 52.9)	82.6 (64.7–132)	0	233 (±173, 74)	200 (131–276)	0	512 (±259, 50.7)	496.8 (401–602)	0	1167 (±508, 43.6)	1023 (841–1310)
8 h	0	46.8 (±23, 49.1)	38.2 (34.6–51.8)	0	71.6 (±33.8, 47.2)	78.3 (43.9–86.7)	0	166 (±60.3, 36.4)	148 (125–208)	0	319 (±118, 37.1)	303.5 (241–359)	0	917 (±582, 63.4)	855 (467–1040)
**Δ9-THCV**															
Predose	15	0 (±0, --)	0 (0–0)	14	0 (±0, --)	0 (0–0)	14	0 (±0, --)	0 (0–0)	14	0 (±0, --)	0 (0–0)	15	0 (±0, --)	0 (0–0)
0.5 h	15	0 (±0, --)	0 (0–0)	14	0 (±0, --)	0 (0–0)	14	0 (±0, --)	0 (0–0)	14	0 (±0, --)	0 (0–0)	15	0 (±0, --)	0 (0–0)
1 h	15	0 (±0, --)	0 (0–0)	14	0 (±0, --)	0 (0–0)	14	0 (±0, --)	0 (0–0)	13	0.1 (±0.3, 374)	0 (0–0)	14	0 (±0.2, 387)	0 (0–0)
2 h	15	0 (±0, --)	0 (0–0)	14	0 (±0, --)	0 (0–0)	14	0 (±0, --)	0 (0–0)	12	0.1 (±0.3, 258)	0 (0–0)	9	0.6 (±0.8, 134)	0 (0–1.5)
4 h	15	0 (±0, --)	0 (0–0)	14	0 (±0, --)	0 (0–0)	14	0 (±0, --)	0 (0–0)	10	0.3 (±0.6, 183)	0 (0–0)	6	0.9 (±1, 112)	0.7 (0–1.5)
6 h	15	0 (±0, --)	0 (0–0)	14	0 (±0, --)	0 (0–0)	13	0.1 (±0.3, 374)	0 (0–0)	10	0.3 (±0.6, 194)	0 (0–0)	7	0.7 (±0.8, 112)	0.6 (0–1.5)
8 h	15	0 (±0, --)	0 (0–0)	14	0 (±0, --)	0 (0–0)	14	0 (±0, --)	0 (0–0)	14	0 (±0, --)	0 (0–0)	9	0.6 (±1.1, 186)	0 (0–0.7)
**Δ9-THCV-COOH**															
Predose	15	0 (±0, --)	0 (0–0)	14	0 (±0, --)	0 (0–0)	14	0 (±0, --)	0 (0–0)	14	0 (±0, --)	0 (0–0)	15	0 (±0, --)	0 (0–0)
0.5 h	15	0 (±0, --)	0 (0–0)	14	0 (±0, --)	0 (0–0)	14	0 (±0, --)	0 (0–0)	12	0.3 (±0.7, 257)	0 (0–0)	14	0.1 (±0.6, 387)	0 (0–0)
1 h	14	0.1 (±0.3, 387)	0 (0–0)	9	0.7 (±1.1, 144)	0 (0–1.7)	11	0.6 (±1.3, 204)	0 (0–0)	6	5.8 (±6.9, 118)	1.9 (0–13.7)	7	4.9 (±7, 143)	2.7 (0–7.6)
2 h	12	0.5 (±1.1, 217)	0 (0–0)	6	1.6 (±1.5, 96.6)	1.7 (0–2.9)	8	2.3 (±3.9, 170)	0 (0–2.1)	4	9.2 (±10.2, 111)	8.7 (0.6–11.6)	5	19.1 (±21.6, 113)	9.4 (0–30.7)
4 h	3	1.9 (±1.3, 70.7)	1.8 (1.1–3.1)	0	3.7 (±1.8, 48.1)	3.6 (2.5–4.9)	2	5.4 (±4.2, 78.3)	5 (2.4–7.9)	0	16.1 (±11.1, 68.7)	16 (11.7–19)	1	37.1 (±22.4, 60.4)	40 (17.6–57.4)
6 h	6	1.5 (±1.6, 107)	1.3 (0–1.9)	1	3 (±1.6, 53.2)	2.4 (2.1–4.5)	0	6.7 (±3.9, 58.4)	5.9 (4.1–8)	0	15.6 (±6.9, 44.2)	17.5 (11.1–20.1)	0	32.3 (±13.1, 40.4)	28.5 (24.2–35.6)
8 h	6	1.2 (±1.1, 92.5)	1.4 (0–2.1)	2	2.3 (±1.4, 61.7)	2.3 (1.4–3.2)	0	5.4 (±1.9, 36)	5.2 (4.2–6.5)	0	10.8 (±3.7, 34.7)	10.5 (7.7–14.1)	0	29.2 (±16.1, 55.3)	26.3 (17.4–35.5)
**Δ8-THC-COOH**															
Predose	15	0 (±0, --)	0 (0–0)	14	0 (±0, --)	0 (0–0)	14	0 (±0, --)	0 (0–0)	14	0 (±0, --)	0 (0–0)	15	0 (±0, --)	0 (0–0)
0.5 h	15	0 (±0, --)	0 (0–0)	14	0 (±0, --)	0 (0–0)	14	0 (±0, --)	0 (0–0)	14	0 (±0, --)	0 (0–0)	15	0 (±0, --)	0 (0–0)
1 h	15	0 (±0, --)	0 (0–0)	14	0 (±0, --)	0 (0–0)	14	0 (±0, --)	0 (0–0)	14	0 (±0, --)	0 (0–0)	15	0 (±0, --)	0 (0–0)
2 h	15	0 (±0, --)	0 (0–0)	14	0 (±0, --)	0 (0–0)	14	0 (±0, --)	0 (0–0)	14	0 (±0, --)	0 (0–0)	11	0.4 (±0.7, 175)	0 (0–0.5)
4 h	15	0 (±0, --)	0 (0–0)	14	0 (±0, --)	0 (0–0)	14	0 (±0, --)	0 (0–0)	10	0.4 (±0.7, 167)	0 (0–0.8)	6	1.3 (±1.2, 90.5)	1.7 (0–2.2)
6 h	15	0 (±0, --)	0 (0–0)	14	0 (±0, --)	0 (0–0)	14	0 (±0, --)	0 (0–0)	8	0.6 (±0.7, 124)	0 (0–1.2)	2	1.8 (±1, 55.2)	2 (1.3–2.4)
8 h	15	0 (±0, --)	0 (0–0)	14	0 (±0, --)	0 (0–0)	14	0 (±0, --)	0 (0–0)	13	0.1 (±0.3, 374)	0 (0–0)	3	1.6 (±1, 62.6)	1.6 (1.1–2.2)

Abbreviations: Δ8-THCV: Δ8-tetrahydrocannabivarin, 11-OH-Δ8-THCV: (±)-11-hydroxy-Δ8-THCV, Δ8-THCV-COOH: 11-nor-9-carboxy-Δ8-THCV, Δ9-THCV: Δ9-tetrahydrocannabivarin, Δ9-THCV-COOH: 11-nor-9-carboxy-Δ9-THCV, Δ8-THC-COOH: (+)-11-nor- Δ8-THC-9-carboxylic acid, BLOQ: number of samples below the lower limit of quantification, SD: standard deviation, %CV: coefficient of variation, IQ Range: interquartile range.
